# Moderate maternal nutrient restriction alters type II alveolar epithelial cell density in the non‐human primate fetal lung

**DOI:** 10.1113/EP093319

**Published:** 2026-03-19

**Authors:** Mitchell C. Lock, Hillary F. Huber, Cun Li, Sandra Orgeig, Peter W. Nathanielsz, Janna L. Morrison

**Affiliations:** ^1^ Early Origins of Adult Health Research Group School of Pharmacy and Biomedical Sciences, Adelaide University Adelaide South Australia Australia; ^2^ Southwest National Primate Research Center Texas Biomedical Research Institute San Antonio Texas USA; ^3^ Department of Animal Science University of Wyoming Laramie Wyoming USA; ^4^ College of Health School of Pharmacy and Biomedical Sciences, Adelaide University Adelaide South Australia Australia

**Keywords:** baboon, fetus, lung, pregnancy, surfactant, undernutrition

## Abstract

Restriction of fetal substrate supply has an adverse effect on surfactant maturation in the lung and thus affects the transition from in utero placental oxygenation to pulmonary ventilation *ex utero*. However, the consequences of reduced fetal substrate supply are dependent on the timing of gestation, severity and duration. We hypothesise that maternal nutrient restriction (MNR) from early pregnancy negatively impacts fetal lung maturation. Female baboons of similar age and weight were randomly assigned to either a control diet (*n* = 3F, 5M offspring) or MNR (*n* = 4F, 4M offspring). On a weight‐adjusted basis, MNR animals were fed 70% of the feed consumed by controls. Fetal lung tissue was collected at 0.9 gestation (term = 184 days). qRT‐PCR and immunohistochemistry were utilised to measure expression of key molecules involved in surfactant maturation, reabsorption of lung liquid, vascularisation and immune cells. MNR decreased type II alveolar epithelial cell density and the mRNA expression of *PCYT1A*, the gene for choline‐phosphate cytidylyltransferase A, the enzyme required for *de novo* surfactant phospholipid synthesis. However, MNR had no effect on the expression of surfactant proteins in the fetal lung. There was a reduced number of α‐smooth muscle actin‐stained vessels and presence of CD45^+^ immune cells within the lung of fetuses exposed to MNR. These data indicate that MNR from early pregnancy increases risk of neonatal respiratory complications at birth by impairing the capacity for surfactant maturation, reducing vascularisation within the fetal lung and impairing innate lung immunity.

## Abstract (highlights)

- **What is the central question of this study?**
  Maternal nutrient restriction during pregnancy is associated with a higher rate of neonatal respiratory distress syndrome: what are the molecular mechanisms behind this?
- **What is the main finding and its importance?**
  Moderate maternal nutrient restriction (70% of control diet) in the baboon model of pregnancy from day 30 to 165 of gestation (term = 184 days) resulted in a reduction of type II alveolar epithelial cells, CD45^+^ immune cells and reduced vascularisation within the fetal lung. These data indicate that maternal nutrient restriction during pregnancy may result in an increased risk of neonatal respiratory distress syndrome via impaired surfactant maturation, innate pulmonary immunity and reduced vascularisation in the fetal lung.

## 1 INTRODUCTION

Despite substantial progress in reducing global poverty and food insecurity in the past 50 years, the prevalence of maternal undernutrition in low‐income and middle‐income countries has remained unacceptably high (Victora et al., 2021), correlating with poor neonatal outcomes and childhood respiratory disease. Substrate supply in utero plays a critical role in the trajectory of fetal growth and development. When supply of nutrients is suboptimal, the fetus cannot reach its genetic growth potential, causing both structural and epigenetic changes that affect fetal organs in different ways (Belkacemi et al., 2010; Karadag et al., 2009; Wu et al., 2004). Optimal development of the fetal lung is critical for neonatal survival but is often sacrificed when substrate supply is limited (McGillick et al., 2025a, b), negatively impacting the transition to air‐breathing (Maritz et al., 2005). Reduced substrate supply can occur through a number of mechanisms, such as placental insufficiency, which results in a reduction in both glucose and oxygen supply to the fetus, a state that impairs lung development (McGillick et al., 2017c; Orgeig et al., 2010). Another common mechanism of reduced substrate delivery is through maternal undernutrition, where there is no change in oxygen delivery but reduced delivery of nutrients (Darby et al., 2018; Ren et al., 2021; Soo et al., 2012). Our previous work has shown that there is little impact of undernutrition isolated only to late gestation on the fetal lungs (Ren et al., 2021); however, the timing, severity and duration of insults are of critical importance in fetal development (Darby et al., 2020; Morrison, 2008).

Possibly the most critical step of fetal lung development is the maturation of the surfactant system in preparation for the transition to air breathing, occurring during the saccular and alveolar phases during late gestation (Hallman et al., 2001). Pulmonary surfactant is a complex mixture of both phospholipid and proteins synthesised by type II alveolar epithelial cells (AECs) and is essential for the reduction of surface tension at the air–liquid interface, maintaining alveolar stability (Orgeig et al., 2015; Rooney, 2001). Reduced oxygen and glucose delivery during development not only causes structural changes to the fetal lung but can also alter surfactant maturation where effects differ depending on the timing and type of insult (McGillick et al., 2017c). For example, the removal of endometrial caruncles in sheep prior to pregnancy, resulting in placental restriction and a reduction of both oxygen and glucose delivery to the fetus throughout gestation, results in reduced surfactant maturation (Orgeig et al., 2010). On the other hand, isolation of the insult to hypoxia only by lowering maternal inspired oxygen from gestational day 105–138 in sheep (term = 150 days) conversely increases surfactant maturation in late gestation (Lock et al., 2023; McGillick et al., 2017b). Other models of placental insufficiency during late gestation such as umbilico‐placental embolisation and single umbilical artery ligation (from 120–140, 104–125 and 108–115 days’ gestation, respectively) exhibit variability in outcomes with either no impact, an increase or decrease in surfactant protein (SFTP) expression depending on the timing of exposure (Cock et al., 2001; Malhotra et al., 2019; Sutherland et al., 2012). These data therefore indicate that the timing, severity and duration of substrate reduction may be the most powerful modulators of surfactant maturation.

Limited evidence exists for an impact of nutrient restriction alone during pregnancy on lung maturation, and the evidence is usually limited to late gestation only. Maternal undernutrition in the absence of fetal hypoxaemia during late gestation is associated with reduced postnatal lung weight in sheep and rats (Chen et al., 2004; Gao et al., 2009) as well as decreased surfactant phospholipids in rats (Chen et al., 2004) but unchanged SFTP expression in sheep (Ren et al., 2021). However, maternal undernutrition earlier in pregnancy (28–77 days’ gestation) increased glucocorticoid receptor (*GR*) and *HSD11B2* mRNA expression in neonatal sheep lungs (Whorwood et al., 2001), indicating a role of substrate supply in glucocorticoid availability and signalling, which again highlights the crucial role of timing. Intracellular glycogen stores are an important precursor for *de novo* synthesis of surfactant phospholipids (Agassandian & Mallampalli, 2013; Batenburg, 1992). On the other hand, increased substrate supply has also been shown to disrupt lung maturation, with hyperglycaemia and hyperinsulinaemia negatively regulating surfactant production during late gestation and counteracting the stimulatory effects of glucocorticoids on the surfactant system (Lock et al., 2017; McGillick et al., 2014, 2017a).

Taken together, the effects of nutrient restriction on fetal lung development appear dependent on the timing, in terms of both onset and duration of the insult. Some data exist for late gestation undernutrition; however, pregnant women are unlikely to reduce substrate supply during late gestation only. Therefore, there is a gap in knowledge of the impact of nutrient restriction on lung maturation from early pregnancy onwards. We hypothesised that moderate reduction in maternal nutrient intake from early pregnancy would negatively impact fetal lung maturation in a clinically relevant model of human pregnancy.

To test this hypothesis, we made use of baboon (*Papio hamadryas* spp.) tissues from a long‐running project investigating outcomes of moderate maternal nutrient restriction (MNR) during pregnancy and lactation. The baboon model of MNR, achieved by feeding mothers 70% of the ad libitum diet 30 days after conception and throughout pregnancy and lactation, results in offspring that are smaller in several morphometric measurements both prenatally and through early postnatal life, with catch‐up growth occurring after weaning (Li et al., 2017). MNR alters the fetal hypothalamo–pituitary–adrenal (HPA) axis, with elevated fetal adrenocorticotropin hormone (ACTH) early in gestation and cortisol later in gestation, indicating programming of the stress axis (Li et al., 2009; Nathanielsz et al., 2020). MNR also disrupts neuronal and glial maturation by reducing cell proliferation and increasing proliferative cell death, impairing formation of neuronal processes and myelinogenesis primarily in the cerebral cortex (Antonow‐Schlorke et al., 2011). In the fetal liver, MNR reduces expression of key insulin‐like growth factor (IGF) system components – IGF‐I, IGF‐II, and their receptors – while increasing IGF binding proteins, which correlates with increased apoptosis and decreased nutrient sensing signals (Li et al., 2009). These physiological and molecular alterations program long‐term changes in metabolic (Choi et al., 2011; Kuo et al., 2018b; Salmon et al., 2018), cardiovascular (Kuo et al., 2017; Kuo et al., 2018a) and neurological systems (Franke et al., 2017; Huber et al., 2015; Keenan et al., 2013).

## 2 METHODS

### 2.1 Ethical approval

All experimental protocols were approved by the Texas Biomedical Research Institute and University of Texas Health Science Center at San Antonio Institutional Animal Care and Use Committees, conducted at the Southwest National Primate Research Center (AAALAC International approved facility) and followed the United States Animal Welfare Act. All animal procedures were performed by fully certified medical doctors or doctors of veterinary medicine (Protocol no. 1134 PC). The tissue used for these studies were part of a pre‐existing biobank. The animals have been studied extensively (Abu Shehab et al., 2014; Antonow‐Schlorke et al., 2011; Cox et al., 2013; Gandhi et al., 2019; Hellmuth et al., 2016; Kakadia et al., 2020; Kakadia et al., 2021; Kamat et al., 2011; Li et al., 2013a; Li et al., 2013b; Li et al., 2013c; Li et al., 2017; Muralimanoharan et al., 2017; Nijland et al., 2007; Nijland et al., 2010; Pantham et al., 2015; Pantham et al., 2016; Pereira et al., 2015; Pereira et al., 2023; Tchoukalova et al., 2014; Unterberger et al., 2009; Xie et al., 2013; Zimmerman et al., 2023). This study is therefore in line with the 3Rs of animal ethics (Tannenbaum & Bennett, 2015), specifically reduction as no new animals were generated for this paper. The specific protocols for the acquisition, analysis, or interpretation of data within this paper were not pre‐registered. The reporting of animal research in this paper adheres to *Experimental Physiology*’s policies and to the essential ARRIVE guidelines (Kilkenny et al., 2010; Percie du Sert et al., 2020). Baboons were maintained in groups of up to 16 in custom‐built outdoor facilities allowing full socialisation and free movement. Methods on the housing of animals, cage design, avoidance of confounding variables, exclusion criteria and environmental enrichment have been reported previously (Schlabritz‐Loutsevitch et al., 2004).

### 2.2 Nutritional diet

Once stable groupings were confirmed, all baboons were fed ad libitum until pregnancy was confirmed. After confirmation of singleton pregnancy at 30 days’ gestation, 16 baboons of similar age (∼11 years of age) and weight were randomly assigned to either a control diet (*n* = 8; *n* = 3 female (F), 5 male (M) offspring) or MNR (*n* = 8; *n* = 4F, 4M offspring); offspring sex was unknown prior to delivery. One single fetus from each baboon pregnancy was considered an experimental unit. On a weight‐adjusted basis, MNR animals were fed 70% of the feed consumed by Controls. Food was provided once a day as Purina Monkey Diet 5LEO, standard biscuits. The biscuit is described as a ‘complete life‐cycle diet for all Old‐World Primates’ and contains stabilised vitamin C and all other required vitamins. The basic composition includes crude protein (≥15%), crude fat (≥4%), crude fibre (≤10%) and ash (≤6.4%) (Choi et al., 2011; Li et al., 2017; Schlabritz‐Loutsevitch et al., 2004).

### 2.3 Post‐mortem and tissue collection

At 165 days’ gestation (term ∼184d), caesarean sections were performed, and the fetus was exteriorised from the uterus and humanely killed by exsanguination while under anaesthesia, a method approved by the American Veterinary Medical Association. Detailed methods on caesarean procedures and post‐operative care and recovery period of female baboons are as previously described (Cox et al., 2006). Briefly, baboons were pre‐medicated with ketamine hydrochloride intramuscularly (10 mg kg^−1^), intubated and maintained at a surgical plane of anaesthesia with isoflurane (2%) throughout the surgery. Anaesthesia was maintained with a mixture of O_2_ and compressed air (2.5 L/min of air, 0.5 L/min of O_2_) and CO_2_ at 35–45%. Depth of anaesthesia was assessed by lack of palpebral reflex, corneal reflex and pain reflex in response to pinching the nail bed to create a pain response. The umbilical cord was used for fetal exsanguination with maternal and fetal baboons under general anaesthesia. Fetal death was confirmed by lack of vital signs, breathing, corneal reflex and response to a firm toe pinch. Fetal lung samples from the lower lobe were collected and snap‐frozen in liquid nitrogen less than 1 h *post mortem* and stored at −80°C for quantitative real‐time PCR or immersion fixed in 4% paraformaldehyde for immunohistochemical analysis.

After surgery, immediate pain management included buprenorphine sustained‐release (0.2 mg/kg i.m.) or daily buprenorphine (0.015 mg/kg/day s.c.) for 3 days. Ketorolac (1 mg/kg i.m.) may be given as an additional analgesic at the conclusion of surgery. Antibiotic prophylaxis commonly consists of ampicillin (25 mg/kg i.m., BID for 7 days) or cephalexin (25 mg/kg p.o. twice daily for 5 days). Mothers were housed individually for postoperative monitoring and recovery, and checked twice daily for pain, wound integrity, posture, mobility, appetite and stool. Technicians specifically examined the incision site for redness, swelling, or discharge. Baboons were returned to their social group cages about 2 weeks after surgery, once the incision was healed and the animal was fully ambulatory with a return to pre‐procedure behaviour, with continued social enrichment and observation.

### 2.4 Real‐time PCR for target genes

All essential information regarding the qRT‐PCR procedure is included as per the MIQE guidelines (Bustin et al., 2009). Total RNA was extracted from frozen lung tissue for each fetus using QIAzol Lysis Reagent solution and QIAgen miRNeasy purification columns, as per the manufacturer's guidelines (Qiagen, Hilden, Germany). Total RNA was quantified by spectrophotometric measurements at 260 and 280 nm in a NanoDrop Lite Spectrophotometer (Thermo Fisher Scientific, Waltham, MA, USA) and the integrity of the RNA was determined using agarose gel electrophoresis. The 260/280 nm ratio results were less than 2.1 and greater than 1.9 and therefore acceptable for qRT‐PCR. cDNA was synthesised using Superscript III First Strand Synthesis System (Thermo Fisher Scientific) using 1 µg of total RNA, random hexamers, dNTP, dithiothreitol and Superscript III in a final volume of 20 µL, as per the manufacturer's guidelines in a MiniAmp Plus Thermal Cycler (Thermo Fisher Scientific). Controls containing either no RNA transcript or no Superscript III were used to test for reagent contamination and genomic DNA contamination, respectively. The geNorm component of qbaseplus 3.4 software (Biogazelle, Ghent,Belgium) was used to determine the most stable reference genes from a panel of candidate reference genes (Vandesompele et al., 2002) and the minimum number of reference genes required to calculate a stable normalisation factor, as previously described (Lie et al., 2014; McGillick et al., 2013; Soo et al., 2012). For qRT‐PCR data output normalisation, two stable reference genes, *TBP* and *ACTB* (Table 1), were run in parallel with all target genes, as previously described (Lock et al., 2017). A selection of genes were chosen a priori to investigate key pathways involved in lung development. Primers were designed, validated and optimised for baboon lung tissue as previously described (Orgeig et al., 2010). Relative expression of target genes (Table 1) involved in surfactant protein and phospholipid production (*SFTPA1*, *SFTPB*, *SFTPC*, *SFTPD*, *PCYT1A*, *ABCA3*, *LPCAT1*), surfactant protein regulatory genes (*TTF1*, *SP1*), glucocorticoid signalling and conversion (*HSD11B1*, *HSD11B2*), water and sodium movement (*AQP1*, *AQP2*, *AQP3*, *AQP4*, *ATP1A1*, *SCNN1B*), glucose transport (*GLUT1*, *GLUT4*), peroxisome proliferator‐activated receptor γ (PPARγ) signalling (*LEPR*, *PPARG*, *PPARGC1A*), fatty acid metabolism (*FASN*, *SLC27A1*) and inflammatory and immune cell markers (*RELA*, *TNF*, *CD4*, *CD7*, *CD45*, *CD68*) were measured by qRT‐PCR using KiCqStart SYBR Green qPCR ReadyMix (Sigma Aldrich, St Louis, MO, USA) in a final volume of 6 µL on a QuantStudio 7 Pro Real‐time PCR system (Thermo Fisher Scientific), as previously described (Lock et al., 2017). Each qRT‐PCR well contained 3 µL SYBR Green Master Mix (2×), 2 µL of forward and reverse primer mixed with H_2_O to obtain final primer concentrations and 1 µL of diluted cDNA. Each sample was run in triplicate for target and reference genes. The abundance of each transcript relative to the abundance of stable reference genes (Hellemans et al., 2007) was calculated and expressed as mRNA mean normalised expression (MNE) ±SD.

**TABLE 1:** qRT‐PCR primer list.

Primer name	Gene symbol	Reference/primer sequence 5′ to 3′	Accession no.
Reference genes			
TATA‐box binding protein	*TBP*	Fwd: GCTTCAGAGAGTTCTGGGATTG Rev: GTGGTTCGTGGCTCTCTTATC	XM_009206461.4
Actin beta	*ACTB*	Fwd: GTCCACCGCAAATGCTTCTA Rev: CACCTTCACCGTTCCAGTTT	XM_003895688.4
Surfactant maturation			
Surfactant protein A1	*SFTPA1*	Fwd: CCTCCAGATGTGACCAGATTAG Rev: CTAAGACCTGGCACACGATAA	XM_003903711.5
Surfactant protein B	*SFTPB*	Fwd: GGATCTCTCTGAGCAGCAATTC Rev: TCGAGCAGGATGACAGAGTAG	XM_009184574.4
Surfactant protein C	*SFTPC*	Fwd: GTCTCCACATGAGCCAGAAA Rev: GCCGCTGGTAGTCATACAC	XM_003902511.3
Surfactant protein D	*SFTPD*	Fwd: GAGCATGACTGACTCCAAGATG Rev: CAAGCCCTGTCATTCCACTT	XM_003903708.4
Phosphate cytidylyltransferase 1, choline, alpha	*PCYT1A*	Fwd: CAACACAGAGGACAGAAGGTATC Rev: CCTCTCCTGCAAGTGGTATTT	XM_009202040.4
ATP‐binding cassette sub‐family A member 3	*ABCA3*	Fwd: CTGCTGGACTCTGTGCTTTAT Rev: CTTTCTCAGGGTCACTGTCTTC	XM_003916379.4
Lysophosphatidylcholine acyltransferase 1	*LPCAT1*	Fwd: CCTCATGACACTGACACTCTTC Rev: CTTCAGCAGGAAGTCCACAA	XM_003899463.4
Surfactant protein regulatory genes			
Sp1 transcription factor	*SP1*	Fwd: CCAATGAGAACAGCAACAACTC Rev: CACTCTGTTCCTTTGAGGTAGG	XM_031650504.1
Transcription termination factor 1	*TTF1*	Fwd: CTTTGGGAGTGAGAAGATGGAG Rev: TCAGAAACTGGAGTGTGGATTT	XM_031654672.1
Glucocorticoid signalling and conversion			
Hydroxysteroid 11‐beta dehydrogenase 1	*HSD11B1*	Fwd: GGCTGGGAAAGTGGCTTAT Rev: ATGGCTGTGTCTGTGTCTATG	XM_009188051.3
Hydroxysteroid 11‐beta dehydrogenase 2	*HSD11B2*	Fwd: TGACCAAACCAGGAGACATTAG Rev: TCCATGCAGCTACGGAAAG	XM_003917042.3
Water and sodium movement			
Aquaporin 1	*AQP1*	Fwd: CGTGGAGGAGGTGAAAGAAA Rev: CCATGACCAAAGGCACAAAG	XM_009203156.3
Aquaporin 2	*AQP2*	Fwd: CCAGCCTGCTGTGTAAGTAATA Rev: TGGAAGCAGAAAGGAGAAAGAG	XM_009180698.4
Aquaporin 3	*AQP3*	Fwd: ACCGAGCAAGAGAATGTGAAG Rev: CCTTAGCCTGGAAGGGTAGAT	XM_003911599.4
Aquaporin 4	*AQP4*	Fwd: GGTGTGCACCAGGAAGATAA Rev: CCATGACCAGCGGTAAGATT	XM_003914257.4
ATPase Na^+^/K^+^ transporting subunit alpha 1	*ATP1A1*	Fwd: GTGTTCCTGGGTGTGTCTTT Rev: ATGCGTTTGGCGGTAAGT	XM_021922594.2
Sodium channel epithelial 1 beta subunit	*SCNN1B*	Fwd: TGGTGTGGTACTGCGATAAC Rev: GCTCAAGTAGGTCCTGATGAAG	XM_009196153.4
Glucose transport			
Glucose transporter 1	*GLUT1*	Fwd: TCCTCATCGCCCAGGTATT Rev: CAGCTTCTTCAGCACACTCTT	NM_001173980.1
Glucose transporter 4	*GLUT4*	Fwd: CCTCAGAAGGTGATTGAACAGA Rev: CCAAGCCACTGAGAGATGATAC	XM_003912229.4
Fatty acid synthesis and transport			
Fatty acid synthase	*FASN*	Fwd: CAACCTGATAGTGAGTGGGAAG Rev: CATGGAAGTGAGGGCCATAG	XM_031657185.1
Solute carrier family 27 member 1	*SLC27A1 (FATP1)*	Fwd: GATCAAGTTCTGCTCTGGAGAC Rev: TGGTCCCTGACGTGTAGAT	XM_009193831.4
PPARγ signalling pathway			
Leptin receptor	*LEPR*	Fwd: CCAACAACTGTGGTCTCTCTAC Rev: CTGATCAGCGTGGCATATTTAAC	XM_031669581.1
Peroxisome proliferator activated receptor gamma	*PPARG*	Fwd: GCACCCTAGGCAAGATCTAAAG Rev: GCATGTGACTATCCCTGTCTTC	XM_021934188.2
PPARG coactivator 1 alpha	*PPARGC1A*	Fwd: TTGACGACGAAGCAGACAAG Rev: CCCAAGGGTAGCTCAGTTTATC	XM_031664083.1
RELA proto‐oncogene, NF‐kB subunit (RELA)	*RELA*	Fwd: CGAGCTTGTAGGAAAGGACTG Rev: AGGGATTGTTGTTCGTCTGG	XM_021925545.2
Tumour necrosis factor (TNF)	*TNF*	Fwd: GCGTAGAGCCGACAGATAAC Rev: TTGACCTTGGTCTGGTAGGA	NM_001112648.1
CD4 molecule (CD4)	*CD4*	Fwd: GCAGTTACCCAGGGAAAGAAA Rev: CTCAGCTTGGATGGACCTTTAG	XM_003905871.4
CD7 molecule (CD7)	*CD7*	Fwd: GGCCTGCATGGGATCTAC Rev: CAGGTGGTGCATGGTAATAGT	XM_003913611.5
Protein tyrosine phosphatase receptor type C (PTPRC)	*CD45*	Fwd: GAGACCACACCATCTCTTAGTTC Rev: CTGCGTGTCAGTTCCAGTAG	XM_021928947.2
CD68 molecule (CD68)	*CD68*	Fwd: CCAACAGTGCCCTACCTTAAT Rev: CACCAGTTGCTCAGCTCTAC	XM_003912264.5

### 2.5 Quantification of Pro‐SFTP‐B, cleaved‐caspase 3, CD45^+^ positive cells and αSMA positive vessels within the fetal lung

For identification of Type II AECs, five different surfactant‐related antibodies were tested for optimal staining in baboon tissue. Specifically, rabbit anti‐human ABCA3 (WRAB‐ABCA3, Seven Hills Bioreagents, Cincinnati, OH, USA), mature surfactant protein (SP)‐B (WRAB‐48604, Seven Hills Bioreagents), mature SP‐C (WRAB‐76694, Seven Hills Bioreagents), pro‐SP‐B (WRAB‐55522, Seven Hills Bioreagents) and pro‐SP‐C (WRAB‐9337, Seven Hills Bioreagents). Following optimisation, immunohistochemistry was then performed using a rabbit anti‐human mature pro‐SFTP‐B (1:300) cleaved‐caspase 3 (1:400, ASP175, Cell Signaling Technology, Danvers, MA, USA), CD45 (1:500, ab10558, Abcam, Cambridge, UK) or anti‐α‐smooth muscle actin (α‐SMA) antibody (1:3000, MA106110, Thermo Fisher Scientific) as previously described (Lock et al., 2015; McGillick et al., 2016a, b). All sections were counterstained with Mayer's haematoxylin (Sigma‐Aldrich). Negative control slides (primary antibody negative control, and rabbit serum negative control) were incubated overnight at 4°C in parallel with test slides under the same experimental conditions as described previously (Lock et al., 2015; McGillick et al., 2016a, b). Stained sections (Control *n* = 8, MNR *n* = 8) were examined using either QuPath (v0.6.0, Bankhead et al., 2017) or Visiopharm new Computer Assisted Stereological Toolbox (NewCAST) software (Visiopharm, Hørsholm, Denmark) as described previously (Lock et al., 2015; McGillick et al., 2016a, b; Amanollahi et al., 2025). Sixty counting frames (×40 magnification) of the alveolar epithelium were randomly allocated per masked tissue section. Positive staining of Type II cells was confirmed with the presence of cuboidal shaped cells exhibiting cytoplasmic staining within the alveolar epithelium of lung tissue sections. Positive vessel staining was identified by 3,3′‐diaminobenzidine (DAB) staining of a continuous ring of smooth muscle in the vascular wall. Point‐counting using an unbiased counting frame with an area of 20,000 µm^2^ (pro‐SPB) or 40,000 µm^2^ (αSMA) on immersion fixed tissue was used to estimate the numerical density of pro‐SFTP‐B positive cells and αSMA positive vessels within the alveolar epithelium of fetal lung tissue sections as described previously (Lock et al., 2015; McGillick et al., 2016a, b). This method of analysis takes into account the number of points of each counting frame falling on lung tissue within each field of view (60 frames × 4 corners = 240 points) and therefore inherently corrects for any differences in lung tissue density between samples.

$$\begin{array}{ccc} & & \mathrm{proSP}-B\;\mathrm{positive}\;\mathrm{cells}\;\mathrm{per}\;mm^{2}\;\mathrm{of}\;\mathrm{lung}\;\mathrm{tissue}=\\ & & \frac{\sum Q^{-}\left(\mathrm{Surfactant}\;\mathrm{positive}\right)}{\sum P\left(\mathrm{Lung}\;\mathrm{tissue}\right)\times \left[\frac{a\left(\mathrm{frame}\right)}{P}\right]}\times 10^{6} \end{array}$$

where Σ*Q*¯ (Surfactant positive) represents the total number of SP‐positive cells counted in all counting frames of one fetal lung tissue section, and Σ*P* (Lung tissue) represents the total number of points falling on lung tissue in each field of view. *P* is the number of points that were used to count the points included within the reference space (four corners per counting frame), and 𝑎 is the total area of the counting frame. Cleaved caspase 3 and CD45^+^ staining was quantified using the QuPath positive cell detection tool with the region of interest covering the entire tissue section. Correct detection of positive cells was confirmed on each slide by an experience user blinded to the treatment groups.

### 2.6 Statistical analysis

All data are presented as means ± standard deviation (SD). Normality of the data was assessed using Shapiro–Wilk test for each measure. All data were normally distributed; thus, the effects of treatment (Control, MNR) and sex were examined using a two‐way analysis of variance (GraphPad Prism version 10, GraphPad Software, Boston, MA, USA). However, as the study was not powered to properly assess effects of sex, and no significant differences were detected, the data were reanalysed using an unpaired Students’ *t*‐test between the Control and MNR groups (GraphPad Prism version 10). Outliers were identified using the ROUT method with a *Q* = 1%. A *P*‐value of <0.05 was considered significant.

## 3 RESULTS

### 3.1 Fetal morphometric measurements

**FIGURE 1:**
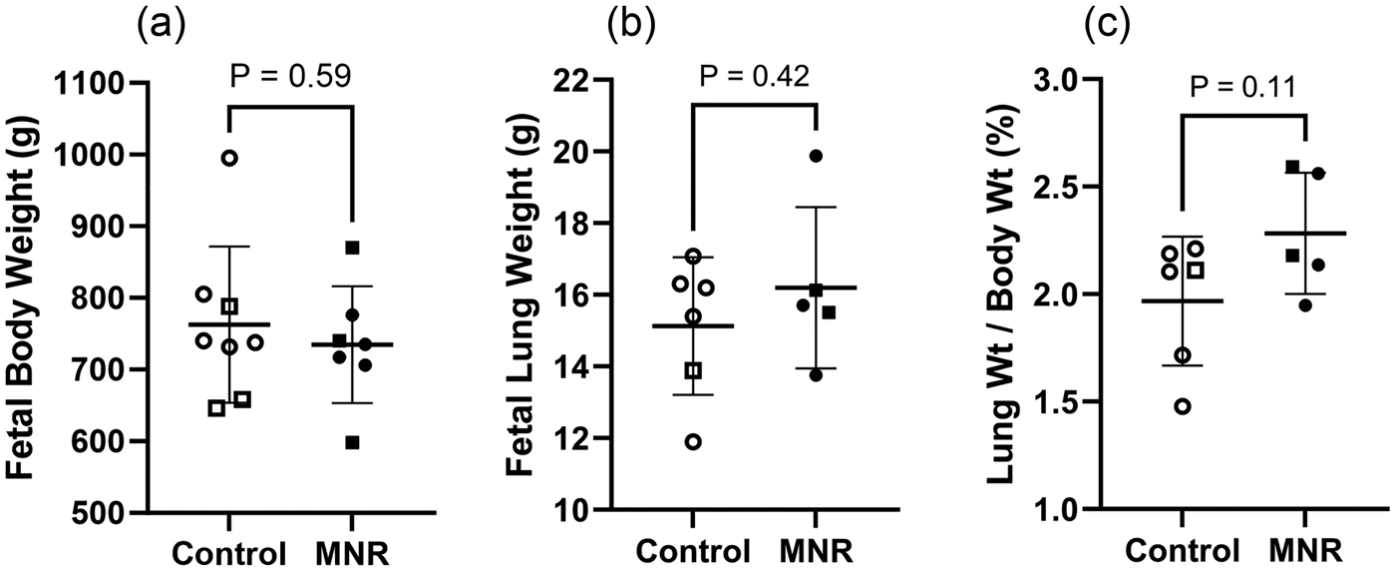
Fetal body parameters. (a) Fetal body weight, (b) fetal lung weight, and (c) lung weight to body weight ratio (expressed as percentage). The effects of treatment were analysed by an unpaired Students’ *t*‐test between the control and MNR groups. *n* = 8 Control (5 male, 3 female), *n* = 8 MNR (4 male, 4 female). *P *< 0.05 was considered significant.

There were no differences in body weight, lung weight or lung to body weight ratio in fetal baboons exposed to MNR compared to controls (Figure 1).

### 3.2 Immunohistochemical analyses

**FIGURE 2:**
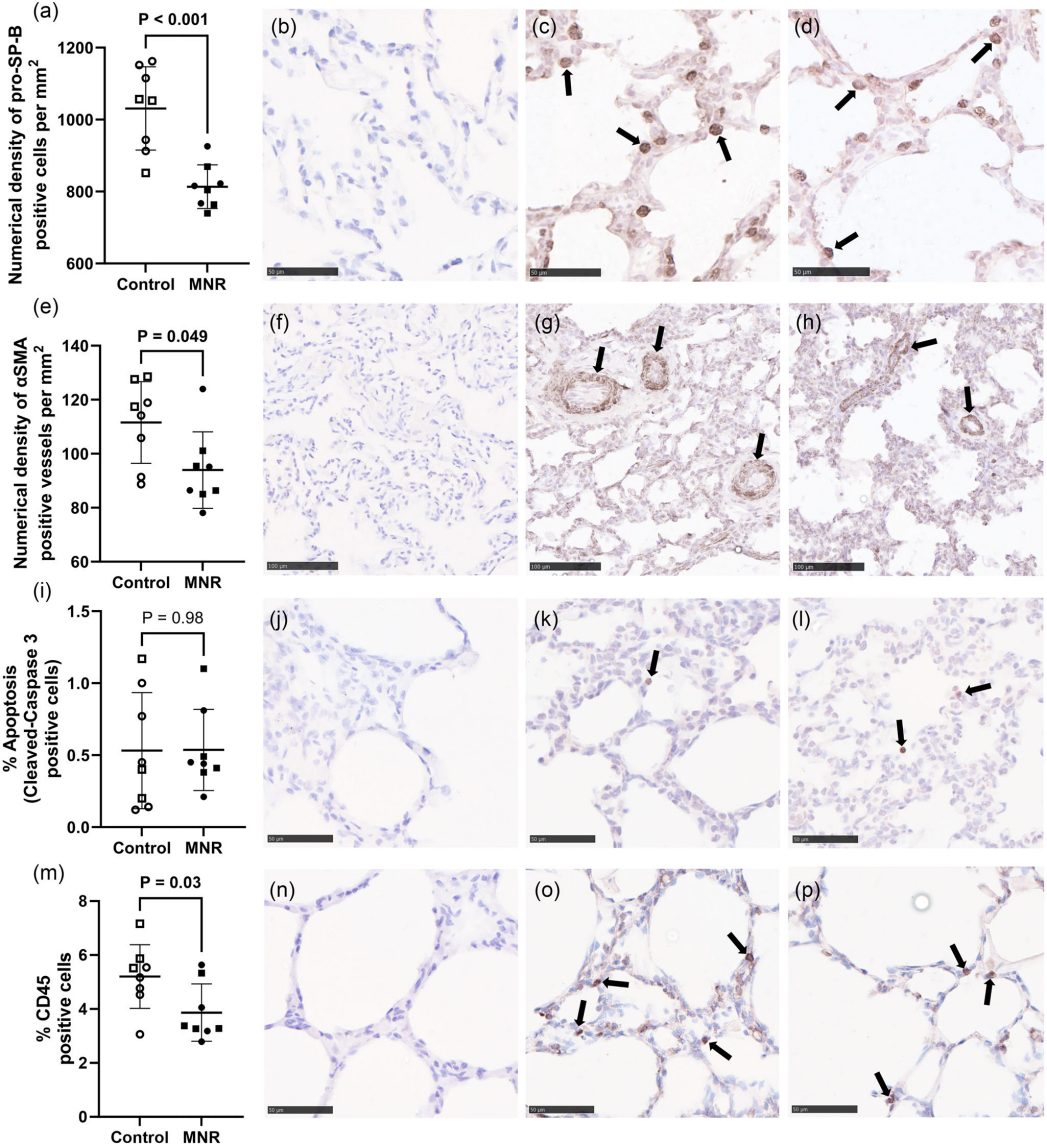
Quantification of type II AECs, vessel density, apoptosis and immune cells in fetal lung tissue. (a) Numerical density of pro‐SP‐B positive cells in the alveolar epithelium. (b–d) Representative micrograph of negative control (b) and pro‐SP‐B staining from control (c) and MNR (d) fetal lung tissue. (e) Numerical density of αSMA stained vessels within the fetal lung. (f–h) Representative micrographs of negative control (f) and αSMA staining from control (g) and MNR (h) fetal lung tissue. (i) percentage of apoptotic cells marked by cleaved‐caspase 3 positive staining. (j–l) Representative micrographs of negative control (j) and cleaved‐caspase 3 staining from control (k) and MNR (l) fetal lung tissue. (m) percentage of immune cells marked by CD45 positive staining. (n–p) Representative micrographs of negative control (n) and CD45 staining from control (o) and MNR (p) fetal lung tissue. The effects of treatment were analysed by an unpaired Students’ *t*‐test between the control and MNR groups. *n* = 8 Control (5 male, 3 female), *n* = 8 MNR (4 male, 4 female). *P *< 0.05 was considered significant.

Immunohistochemical analyses (Figure 2) revealed a decrease in the number of type II AECs within the alveolar epithelium of lungs of fetal baboons exposed to MNR (Figure 2a). There was a decrease in the density of αSMA positive vessels in the MNR group compared to controls (Figure 2e). However, MNR had no effect on the number of cleaved‐caspase 3‐labelled cells undergoing apoptosis within the fetal lung (Figure 2i). CD45 positive cells were reduced in the lungs of MNR fetuses compared to controls (Figure 2m).

### 3.3 mRNA expression analyses

#### 3.3.1 Effect of MNR on markers of surfactant maturation within the fetal lung

**FIGURE 3:**
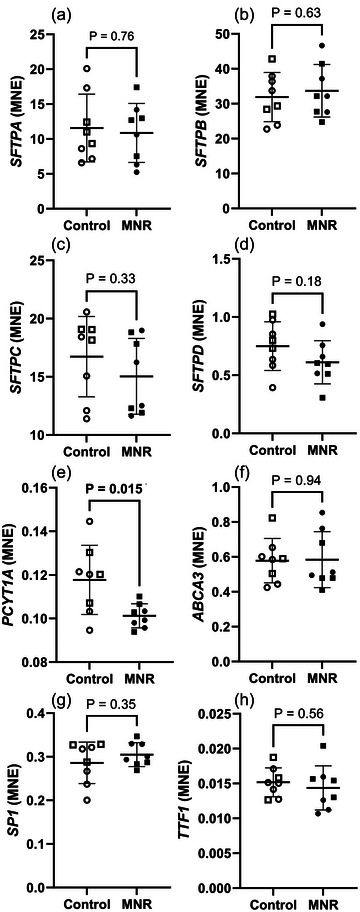
Surfactant maturation markers mRNA expression in control and MNR fetal lungs. (a) *SFTPA*, (b) *SFTPB*, (c) *SFTPC*, (d) *SFTPD*, (e) *PCYT1A*, (f) *ABCA3*, (g) *SP1*, (h) *TTF1*. The effects of treatment were analysed by an unpaired Students’ *t*‐test between the control and MNR groups. *P *< 0.05 was considered significant (bolded values). *n* = 8 Control (5 male, 3 female), *n* = 8 MNR (4 male, 4 female). MNE, mean normalised expression.

Though the number of type II AECs was decreased, there was no change in the expression of genes for surfactant proteins: *SFTPA* (Figure 3a), *SFTPB* (Figure 3b), *SFTPC* (Figure 3c) and *SFTPD* (Figure 3d). There was no change ATP‐binding cassette subfamily A member 3, a vital protein in lung cells, responsible for transporting lipids into lamellar bodies (*ABCA3*, Figure 3f), thyroid transcription factor 1 (*TTF1*, Figure 3h) or surfactant protein regulating transcription factor specificity protein 1 (*SP1*, Figure 3g) in the lungs of MNR fetuses compared to controls. In line with the decreased number of type II AECs, there was a decrease in mRNA expression of *PCYT1A*, encoding choline‐phosphate cytidylyltransferase A, the rate limiting enzyme involved in *de novo* surfactant phospholipid production (Figure 3e), in fetal lungs as a result of MNR.

#### 3.3.2 Effect of MNR on markers of water and sodium movement in the fetal lung

**FIGURE 4:**
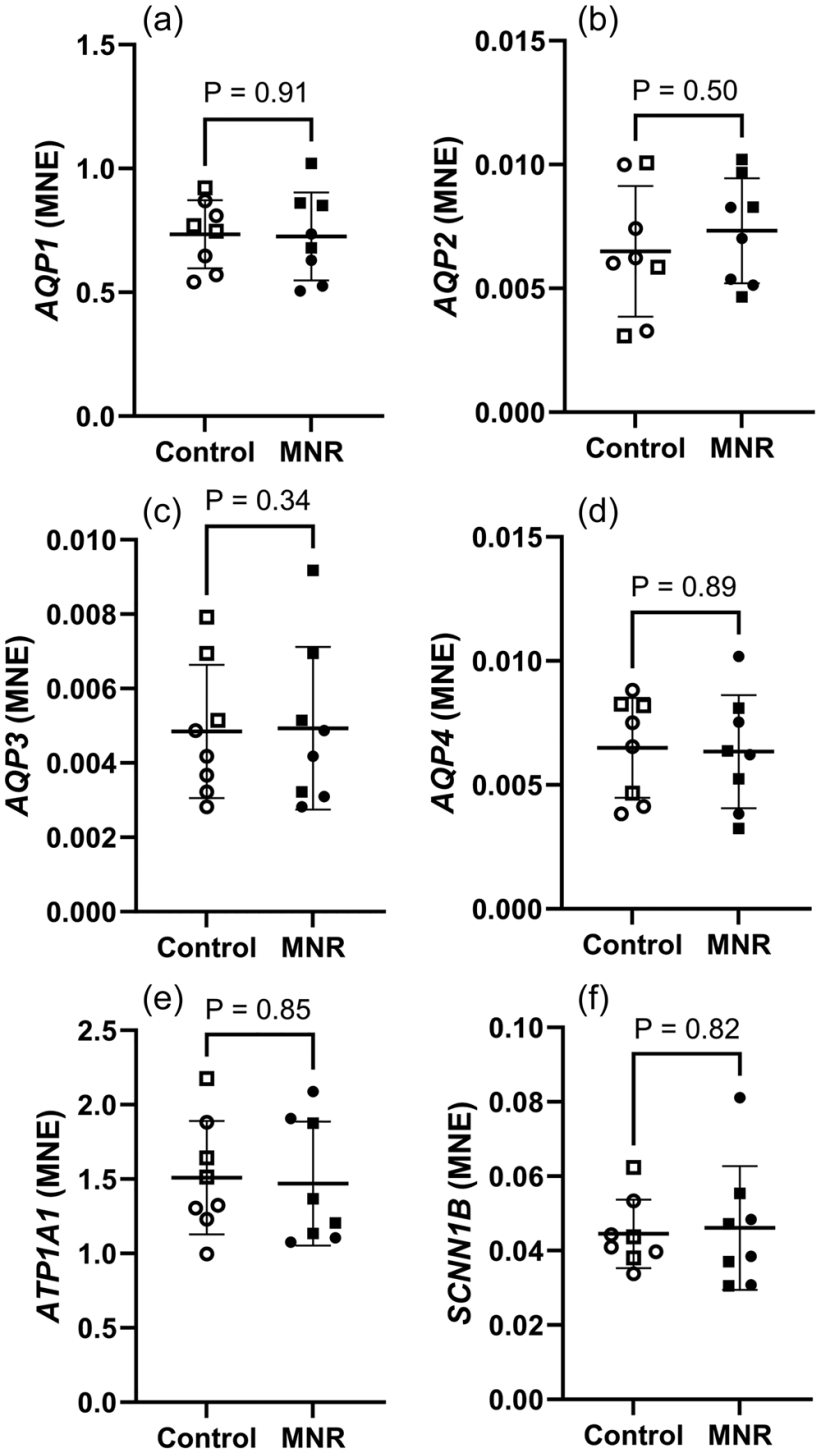
mRNA expression of water and sodium movement markers in control and MNR fetal lungs. (a) *AQP1*, (b) *AQP2*, (c) *AQP3*, (d) *AQP4*, (e) *ATP1A1*, (f) *SCNN1B*. The effects of treatment were analysed by an unpaired Students’ *t*‐test between the control and MNR groups. *n* = 8 Control (5 male, 3 female), *n* = 8 MNR (4 male, 4 female). *P *< 0.05 was considered significant. MNE, mean normalised expression.

There were no changes in the expression of water or sodium transporters *AQP1* (Figure 4a), *AQP2* (Figure 4b), *AQP3* (Figure 4c), *AQP4* (Figure 4d), *ATP1A1* (Figure 4e) or *SCNN1B* (Figure 4f) in fetal lungs in response to MNR.

#### 3.3.3 Effect of MNR on upstream regulators of fetal lung maturation

**FIGURE 5:**
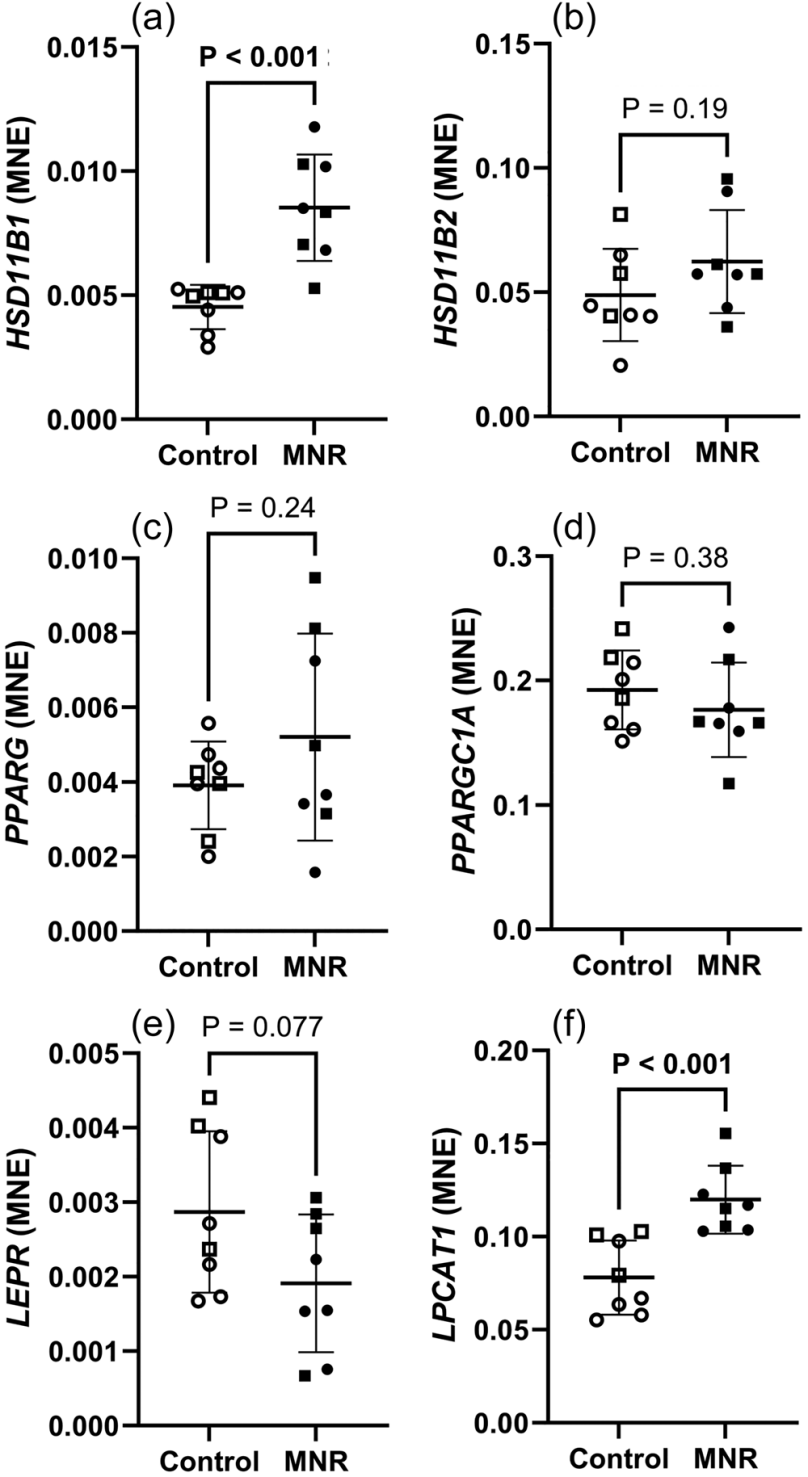
Glucocorticoid conversion and PPARγ signalling mRNA expression in control and MNR fetal lungs. (a) *HSD11B1*, (b) *HSD11B2*, (c) *PPARG*, (d) *PPARGC1A*, (e) *LEPR*, (f) *LPCAT1*. The effects of treatment were analysed by an unpaired Students’ *t*‐test between the control and MNR groups. *n* = 8 Control (5 male, 3 female), *n* = 8 MNR (4 male, 4 female). *P *< 0.05 was considered significant (bolded values). MNE, mean normalised expression.

The mRNA expression of *HSD11B1* (Figure 5a) and *LPCAT1* (Figure 5f) was increased in response to MNR. There was no change in the mRNA expression of *HSD11B2* (Figure 5b) or genes for PPARγ signalling *PPARG* (Figure 5c), *PPARGC1A* (Figure 5d) and *LEPR* (Figure 5e) in the fetal lung in response to MNR.

#### 3.3.4 Effect of MNR on glucose and fatty acid synthesis and transport within the fetal lung

**FIGURE 6:**
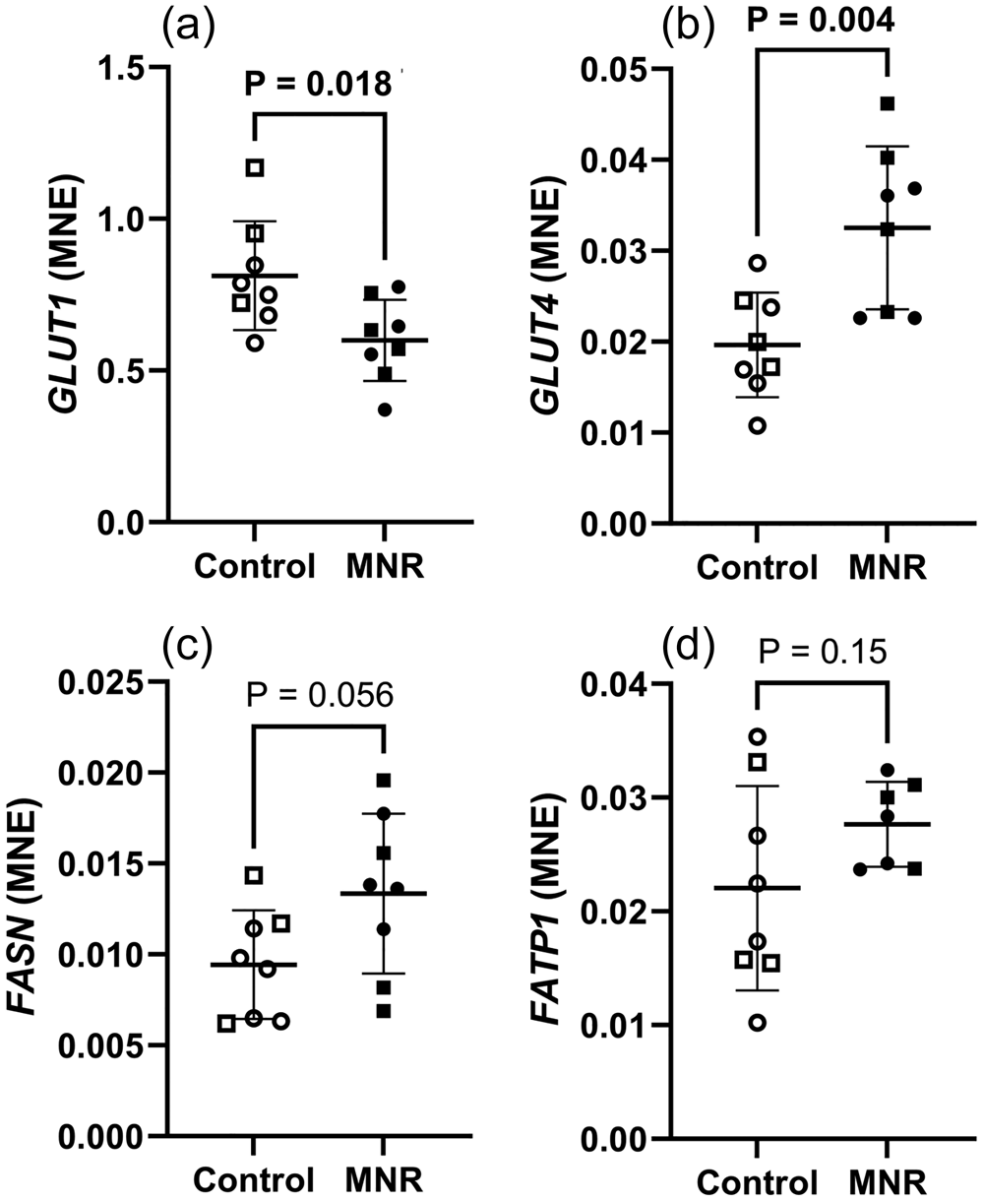
Glucose and fatty acid transport and synthesis mRNA expression in control and MNR fetal lungs. (a) *GLUT1*, (b) *GLUT4*, (c) *FASN*, (d) *FATP1*. The effects of treatment were analysed by an unpaired Students’ *t*‐test between the control and MNR groups. *n* = 8 Control (5 male, 3 female), *n* = 8 MNR (4 male, 4 female). *P *< 0.05 was considered significant (bolded values). MNE, mean normalised expression.

The mRNA expression of *GLUT1* (Figure 6a) was downregulated, whereas *GLUT4* (Figure 6b) was upregulated in the fetal lung in response to maternal MNR. mRNA expression of fatty acid metabolism genes *FASN* and *FATP1* (Figure 6c–d) was unchanged in the fetal lung in response to MNR.

#### 3.3.5 Effect of MNR on immune cells and inflammatory markers within the fetal lung

**FIGURE 7:**
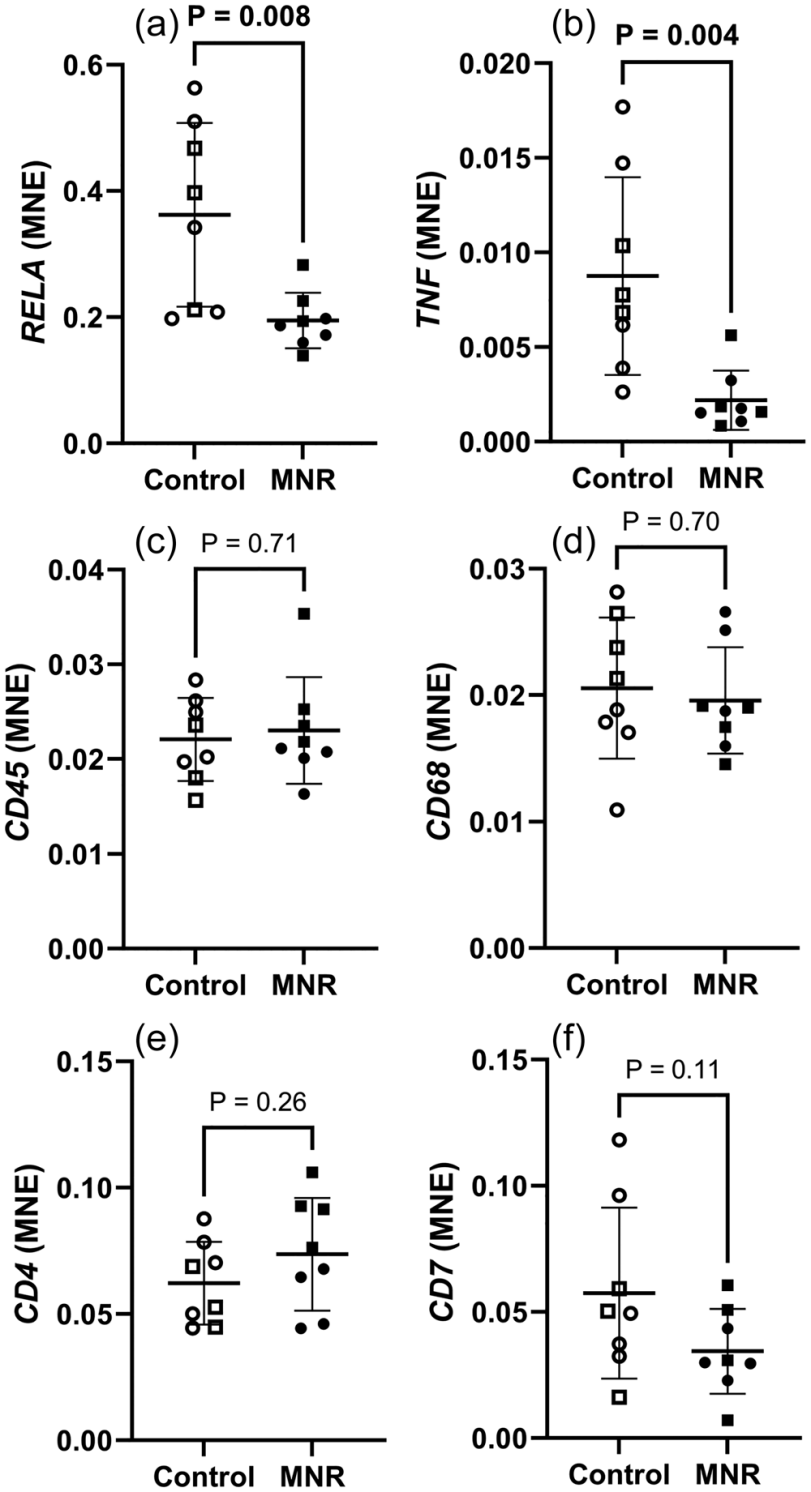
Immune marker mRNA expression in control and MNR fetal lungs. (a) *RELA*, (b) *TNF*, (c) *CD45*, (d) *CD68*, (e) *CD4*, (f) *CD7*. The effects of treatment were analysed by an unpaired Students’ *t*‐test between the control and MNR groups. *n* = 8 Control (5 male, 3 female), *n* = 8 MNR (4 male, 4 female). *P *< 0.05 was considered significant (bolded values). MNE, mean normalised expression.

Though there was a reduction in the number of CD45 positive cells, there was no difference in mRNA expression of immune cell markers *CD45*, *CD68*, *CD4* or *CD7* (Figure 7c–f). mRNA expression of inflammatory markers *RELA* and *TNF* was downregulated in MNR lungs compared to controls, indicating a supressed innate inflammatory environment (Figure 7a–b).

## 4 DISCUSSION

Maternal substrate restriction can impair normal lung maturation, adversely affecting the transition to the air‐breathing environment (Maritz et al., 2005). In this study we utilised a clinically relevant model of moderate MNR from early gestation and throughout pregnancy to determine the impact on fetal lung maturation. Non‐human primates closely mirror the timing of phases of human lung development, offering powerful insight into how the specific window of reduced substrate supply is a driver for the trajectory of fetal lung development. We observed molecular changes in response to MNR measured by qRT‐PCR and immunohistochemistry. Importantly there seems to be a larger structural physiological impact than molecular impairment of lung maturation, indicating that the fetal lung is less physically prepared for the transition to air breathing when exposed to long lasting MNR compared to a control diet, but requires functional validation.

The protocol of moderate MNR utilised within this study offers superior clinical relevance than previous animal studies given that undernutrition begins early in pregnancy and continues throughout (Darby et al., 2018; Ren et al., 2021; Soo et al., 2012). Most previous work has focussed on a late gestation reduction of nutrient intake (Darby et al., 2018; Ren et al., 2021; Soo et al., 2012), which is unlikely to occur in a clinical setting other than rare incidents such as famine (Lumey et al., 2011). Though these studies are useful to determine the impact of nutrient restriction on the saccular and alveolar phases of lung development (a time of rapid acceleration of maturation for the lung), it is important to understand how the lungs are affected if receiving fewer nutrients throughout pregnancy and are therefore behind the normal developmental trajectory. Though we saw no change in fetal body weight, in this cohort of animals, lung weight or lung to body weight ratio (Figure 1), there were clear immunohistological changes that indicate alveolar epithelium structural impairment (Figure 2a), and reduced vascularisation (Figure 2e).

Near term in many mammalian species, the fetal adrenal cortex is activated and becomes the major source of circulating glucocorticoids for the developing fetus, secreting approximately three‐fourths of the circulating cortisol (Beitins et al., 1973). It has been widely demonstrated that this prepartum rise in plasma cortisol concentration aligns with important pulmonary maturational events (Bolt et al., 2001; Tan et al., 1999). Cortisol acts within the fetal lung by increasing type II AEC differentiation and SFTP expression, thinning of the pulmonary mesenchyme, increasing the proportion of alveolar ducts and increasing lung compliance via production of surfactant phospholipids (Ballard et al., 1997; Boland et al., 2004; Flecknoe et al., 2004). The intracellular mediator of cortisol action is GR, which binds to glucocorticoids (GC) to form a GC–GR complex and translocates to the nucleus to interact with glucocorticoid response elements (GRE) present on target genes (Funder, 1993). As SFTP promoter regions do not have GREs, their expression is regulated by a more indirect method, through transcription factors, which contain GREs (Morrison et al., 2012) and can bind to SFTP promoters, such as TTF1/NKX2‐1 and SP1 (Figure 3e and f). We have previously shown that this model of MNR increases both maternal and fetal cortisol concentration at 0.9 gestation (Guo et al., 2013; Li et al., 2017; Zambrano et al., 2014), and therefore expected a response within in the fetal lung to the elevated plasma cortisol concentration.

Glucocorticoid availability during pregnancy is controlled by the regulatory enzyme isoforms, 11β‐hydroxysteroid dehydrogenase (11βHSD)‐1 and 11βHSD‐2 in the placenta, where 11βHSD‐2 acts as a partial barrier, as well as tissue‐specific locations such as the lung, liver and adipose tissue (Burton & Waddell, 1999; Garbrecht et al., 2006). 11βHSD‐1 and ‐2 regulate the bioavailability of cortisol within tissues by catalysing the conversion of the inactive form of cortisone to the active form, cortisol, or the active form cortisol to the inactive form, cortisone, respectively (Tomlinson & Stewart, 2001). The higher expression of *HSD11B1* and phospholipid remodelling/trafficking enzyme *LPCAT1* in MNR lungs may represent a compensatory stress response to promote surfactant production (Figure 5). Supporting this finding, previous studies on fetal MNR in this model also showed increased activity of *HSD11B1* in the fetal liver in males and adipose tissue in female with increased local cortisol concentrations (Guo et al., 2013).

Surfactant proteins synthesised by type II AECs are essential for the spreading of surfactant phospholipids at the hypophase (SFTPB and C) and aiding in the development of innate immunity (SFTPA and D) (Han & Mallampalli, 2015; Orgeig et al., 2015). Interestingly, moderate nutrient restriction had no effect on the mRNA expression of SFTPs. These data are similar to those of previous work investigating late gestation undernutrition in fetal sheep, where there was no difference detected in surfactant protein mRNA or protein expression (Ren et al., 2021). However, there was a reduction in the number of type II AECs, alongside a reduction in *PCYT1A* mRNA expression, the rate limiting enzyme involved in *de novo* production of the phospholipid phosphatidylcholine, indicating a lower capacity for surfactant production in the lungs of MNR fetuses.

Interestingly, *GLUT1* was downregulated in response to MNR, but the insulin‐dependent glucose transporter *GLUT4* was upregulated (Figure 6). This dysregulation is likely in response to the overall reduced glucose availability in the maternal and fetal circulation, with a higher abundance of insulin‐dependent glucose transporter required for uptake of the limited nutrients available. This dysregulation is unlike maternal late gestation overnutrition (150% metabolisable energy requirements) that results in a reduction of surfactant protein expression and expression of both GLUT1 and GLUT4 in the fetal sheep lung, likely linked to elevated insulin, which has an inverse relationship with surfactant production (Lock et al., 2017). We had anticipated that there would be a ‘horseshoe’ like response within the lung, where both extremes of maternal nutrition (high and low levels of substrate) impair surfactant system maturation (Ren et al., 2021); however, it seems that only high levels of nutrients directly impair the production of surfactant proteins during late gestation (Lock et al., 2017).

Potentially the most physiologically important difference within the fetal lung in the current model of MNR is the reduction in type II AEC (Figure 2a). This change may be explained by the fact that although lung maturation rapidly occurs during the saccular and alveolar phases, type II AECs appear in the canalicular phase (Burri, 2006), indicating that undernutrition during this period may have a more substantial effect on the number of these cells. Though it was not measured in previous studies (Ren et al., 2021), αSMA positive vessel density was also lower in MNR, indicating reduced surface area for oxygen transfer within the alveolar epithelium (Figure 2e). Our previous work did not find any difference in pulmonary blood flow using MRI in response to late gestation maternal undernutrition in sheep (Ren et al., 2021), indicating again the importance of timing and severity of the diet intervention as well as possible species specific differences in the response to reduced substrate supply. Our data indicate that there may be no direct effect of blood flow to the lungs; however, maternal undernutrition from early pregnancy may instead impair gas exchange after birth via reduced vascularisation. Indeed, although there were no observed differences in aquaporin or sodium transporter mRNA expression, which are required for the initial reabsorption of lung liquid at birth, the reduced vessel density implies that there may be a more limited rate of transfer into the neonatal circulation.

During development, immune–epithelial interactions contribute to lung maturation and the formation of a healthy respiratory system (Yoshida et al., 2025). One key role of resident innate immune cells is production of the cytokine IL‐1β in driving the differentiation of epithelial tip progenitor cells into airway basal cells (Barnes et al., 2023). At birth, the fetal lung also requires a strong innate immune system to prevent infection after the transition to air breathing (Lambert & Culley, 2017). We detected reduced presence in CD45^+^ immune cells (CD45 being a marker present on all immune cells) in the alveolar epithelium (Figure 2m), as well as a reduction in inflammatory markers *RELA* (a subunit of NF‐κB) and *TNF* in MNR compared to controls (Figure 7), indicating impaired lung innate immune system. To the best of our knowledge, resident lung immune cell deficiency has not previously been reported in the nutrient restricted fetal lung; however, further investigation of expression of cell specific markers (*CD4*, *CD7* and *CD68*) did not reveal which specific cell types are suppressed (Figure 7). Interestingly there was no difference detected in the mRNA expression of *CD45*. This discrepancy may be explained by post‐transcriptional modulation of mRNA expression (for example suppression by miRNAs), the individual cells expressing a higher amount of CD45, or a difference in diversity of immune cells with varying expression of CD45. Fewer resident immune cells present at birth will leave the neonatal lung relatively unprotected against infection and may increase the incidence of neonatal pneumonia, which has been observed in populations with maternal malnutrition (Victora et al., 1999). Direct evidence linking maternal undernutrition to an increased change of neonatal pulmonary infection requires further study.

Lung development was not the original primary outcome of the animal component of the study design; we were therefore unable to assess some key markers of maturation. Bronchoalveolar lavage fluid was not collected, and hence surfactant phospholipid analysis could not be performed. Given the limited tissue available, we chose to extract RNA rather than protein, as this allowed us to examine a greater range of molecular targets. In addition, we have previously demonstrated tight correlation between the expression of markers of surfactant maturation between qRT‐PCR and western blot techniques (Lock et al., 2023; Orgeig et al., 2010). Lung tissue was not inflation‐fixed; therefore we could not assess airspace ratio. Lastly, though sex of the fetuses is represented in the graphs using different symbols, we were not appropriately powered to assess the influence of sex on lung developmental outcomes.

### 4.1 Conclusion

This is the first study to assess the impact of moderate MNR from 0.16 to 0.9 of gestation on lung maturation in a clinically relevant large animal model. We found that fetal baboon lungs at 0.9 of gestation were developmentally impaired by moderate MNR compared to control diet. This included a lower density of type II AECs, lower vascularisation and fewer immune cells present in the alveolar epithelium. Although there was no change in surfactant protein expression, there was reduced expression of the key rate limiting enzyme involved in surfactant phospholipid production. The importance of cortisol in both physiological and pathophysiological development of the fetal lung is again highlighted. Taken together these data imply that moderate MNR throughout pregnancy can impair the trajectory of lung development, particularly structural aspects, resulting in a fetal lung that is less‐equipped for the transition to air‐breathing at birth and therefore may increase the risk of developing neonatal respiratory distress syndrome.

## AUTHOR CONTRIBUTIONS

Study conceptualisation: Mitchell C. Lock, Janna L. Morrison, Peter W. Nathanielsz Experimental design and data acquisition: Mitchell C. Lock, Cun Li, Hillary F. Huber, Janna L. Morrison, Peter W. Nathanielsz Analysis or interpretation of data: Mitchell C. Lock, Sandra Orgeig, Janna L. Morrison, Peter W. Nathanielsz Drafting of the work, construction of the manuscript or revising it critically for important intellectual content: Mitchell C. Lock, Sandra Orgeig, Cun Li, Hillary F. Huber, Janna L. Morrison, Peter W. Nathanielsz All authors approved the final version of the manuscript and agree to be accountable for all aspects of the work in ensuring that questions related to the accuracy or integrity of any part of the work are appropriately investigated and resolved. All persons designated as authors qualify for authorship, and all those who qualify for authorship are listed.

## CONFLICT OF INTEREST

None declared.

## Data Availability

The data generated and analysed during this study are available from the corresponding author on reasonable request.
